# Patent ductus arteriosus, systemic NT-proBNP concentrations and development of bronchopulmonary dysplasia in very preterm infants: retrospective data analysis from a randomized controlled trial

**DOI:** 10.1186/s12887-021-02750-9

**Published:** 2021-06-19

**Authors:** Solomiia Potsiurko, Dmytro Dobryanskyy, Lesya Sekretar

**Affiliations:** Department of Pediatrics, No. 2, Danylo Halytsky Lviv National Medical University, 69 Pekarska St., Lviv, 79010 Ukraine

**Keywords:** NT-proBNP, Prognostic value, Bronchopulmonary dysplasia, hemodynamically significant patent ductus arteriosus

## Abstract

**Background:**

Patent ductus arteriosus (PDA) is a common complication in very preterm infants. It is known that there is an association between PDA and development of bronchopulmonary dysplasia (BPD) or death before the postmenstrual age (PMA) of 36 weeks, but this association remains one of the most controversial aspects of the problem. The study aimed to evaluate the relationship between PDA, serum NT-proBNP levels at 2–3 and 8–9 days of life, and BPD/death in very preterm infants.

**Methods:**

Data of 52 preterm infants with a gestational age < 32 weeks, chronological age < 72 h, and PDA diameter > 1.5 mm, enrolled in a randomized controlled trial, were used for the retrospective analysis. All patients underwent daily echocardiographic and two serum NT-proBNP measurements within the first 10 days after birth. Two groups of infants were formed retrospectively at PMA of 36 weeks depending on the outcome, BPD (*n* = 18)/death (*n* = 7) or survival without BPD (*n* = 27). Receiver operator characteristic (ROC) curve was used to evaluate the predictive performance of serum NT-proBNP levels for BPD/death occurrence.

**Results:**

The percentage of infants who received pharmacological treatment for PDA did not differ between the groups. Based on the area under the ROC curve, serum NT-proBNP levels on the 2–3 day of life (AUC = 0.71; 95% confidence interval (CI): 0.56–0.9; *p* = 0.014)) and on the 8–9 day of life (AUC = 0.76; 95% CI: 0.6–0.9; *p* = 0.002) could reliably predict BPD/death in very preterm infants who had PDA diameter > 1.5 mm in the first 72 h of life. Hemodynamically significant PDA (hsPDA) was significantly more often detected in newborns with BPD/death, however, treatment of infants with hsPDA did not reduce the incidence of BPD/death.

**Conclusions:**

In very preterm infants with PDA > 1.5 mm at the age of 24–48 h, serum NT-proBNP concentration could reliably predict the development of BPD or death, regardless of the persistence of PDA, with the highest diagnostic value at 8–9 days.

**Trial registration:**

This study is registered in ClinicalTrials.gov - NCT03860428 on March 4, 2019.

## Background

Patent ductus arteriosus (PDA) occurs in more than 50% of preterm infants < 28 weeks of gestation [1]. It is known that there is an association between PDA and the risk of bronchopulmonary dysplasia (BPD) developing and/or death before 36 weeks of postmenstrual age (PMA). However, the risk does not depend on either presence or absence of PDA, but on the magnitude of PDA shunt [2, 3]. A large left-to-right PDA shunt leads to increased pulmonary blood flow and alters the pulmonary fluid filtration rate, which can cause pulmonary edema, increased pulmonary pressure, and induce structural changes that may contribute to the development of BPD [4].

A recent study showed that infants < 28 weeks of gestation who were exposed to moderate-to-large PDA shunts for at least 7–13 days had a significantly increased risk of developing BPD or death, and exposure for at least 14–27 days affected the BPD risk alone [5]. However, it is unknown whether this association is causal, as the results of numerous randomized controlled trials using prophylactic or early PDA treatment have not shown a decrease in the incidence of BPD or death [6, 7]. Age at the time of PDA treatment did not significantly affect its effectiveness either [8]. With increased survival rates achieved in recent decades, BPD is diagnosed in about 25% of very-low-birth-weight infants [9]. BPD is a common potentially dangerous disease that worsens the long-term prognosis for health and development [9]. Due to the lack of effective treatment, prevention of this disease is important and it should begin as soon as possible. Therefore, it is very important to predict possible development of BPD in very preterm newborns. The most important risk factor for BPD is gestational age, but almost a third of extremely preterm infants do survive without the disease [10]. The search for additional prognostic criteria that would help to solve this problem is still ongoing. Among such criteria, important attention is paid to biomarkers pathogenetically related to the development of BPD, the advantage of which is objectivity and feasibility. However, the diagnostic value of many tested biomarkers remains insufficient, thus mandating the need for reliable early indicators of BPD development [11].

A recent study by Rodríguez-Blanco et al. showed that N-terminal pro-brain natriuretic peptide (NT-proBNP) level in very preterm infants may be a much more informative indicator for the prediction of BPD than echocardiography, because the elevated levels of NT-proBNP more accurately predicted BPD development than the detection of hsPDA by echocardiography alone [12]. The systematic review and meta-analysis of studies [13] showed that the sensitivity and specificity of NT-proBNP levels in preterm infants ≤32 weeks of gestation in the prediction of BPD or death ranged from 61 to 85% and from 71 to 73%, respectively, and in the prediction of BPD only – from 89 to 100% and from 69 to 86%, respectively. However, the infants’ age at the time of NT-proBNP testing was different in different studies, ranging from the first 48 h of life until PMA of 36 weeks. The included studies differed in methodology, peptide determination methods, prognostic thresholds, gestational age, etc.

We hypothesized that serum NT-proBNP levels may be elevated in very preterm infants at risk of developing BPD/death in the first 10 days after birth, and this association is independent of the presence of PDA. Therefore, our study aimed to evaluate the relationship between PDA, early serum NT-proBNP levels, and BPD/death in very preterm infants < 32 weeks of gestation.

## Methods

### Study design and patients

**Fig. 1:**
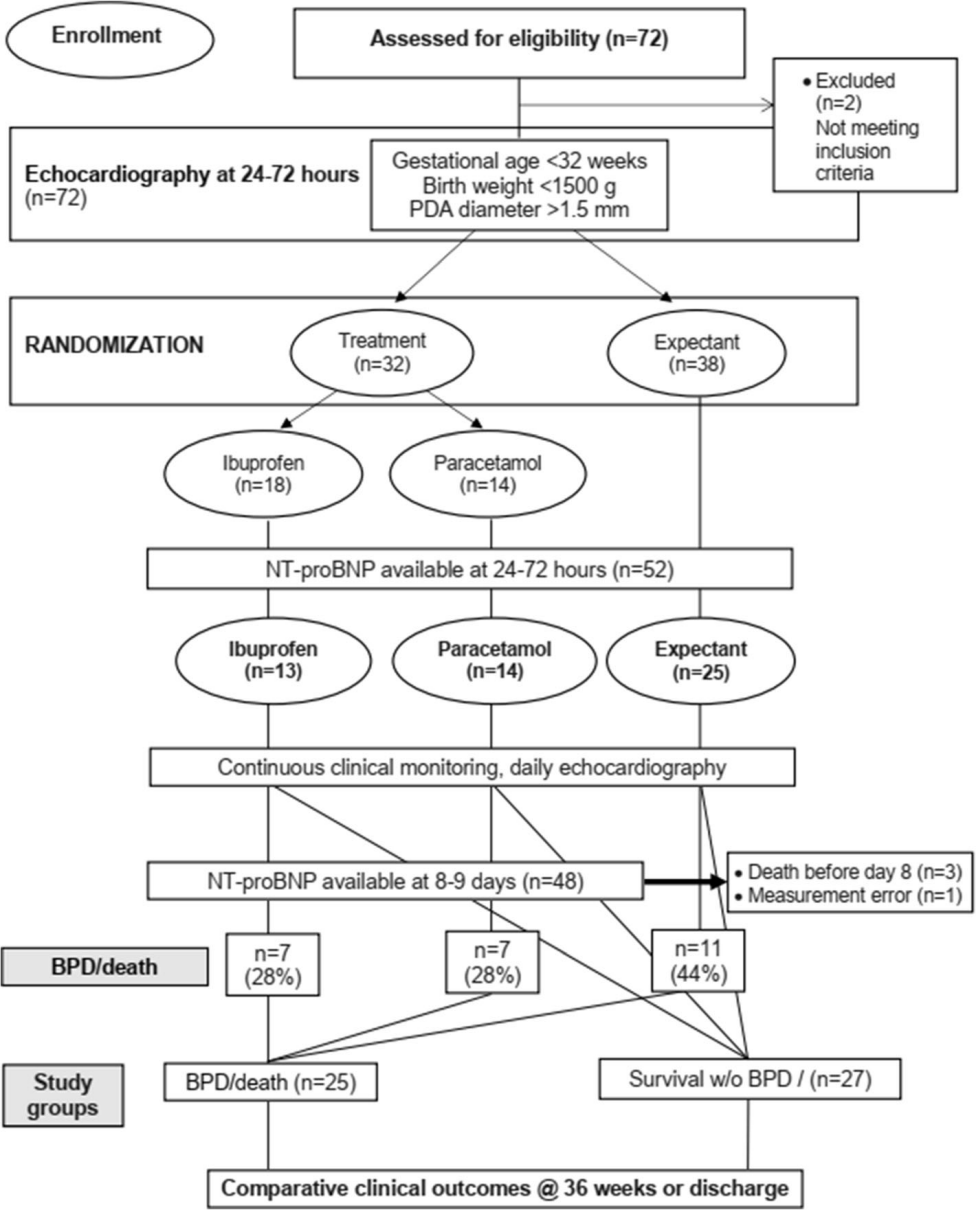
Flow chart of study patients. BPD = bronchopulmonary dysplasia; NT-proBNP = N-terminal pro-brain natriuretic peptide; PDA = patent ductus arteriosus

Data for 52 outborn preterm infants, enrolled in a randomized, controlled, parallel trial to evaluate the comparative efficacy and safety of the early treatment and expectant management of PDA in very preterm infants (*ClinicalTrials.gov* - NCT03860428) between March 2019 and January 2020, were used in this retrospective study (Fig. 1). This study was conducted in the neonatal intensive care unit (NICU) of the Lviv Regional Clinical Hospital, Ukraine. The main trial is currently underway.

Two groups of infants were formed retrospectively at PMA of 36 weeks depending on the outcome, BPD/death (*n* = 25) or survival without BPD (*n* = 27). In the BPD/death group, 18 (72%) infants have survived with BPD, and 7 infants (28%) died.

Inclusion criteria were as follows: birth weight < 1500 g, gestational age < 32 weeks, age at admission to the NICU < 72 h, and PDA diameter > 1.5 mm. Exclusion criteria listed a presence of congenital heart defect other than PDA, clinically apparent haemorrhagic syndrome, any intraventricular haemorrhage (IVH) in the first 48 h or IVH grade 3–4 at any time, suspected/apparent necrotizing enterocolitis (NEC), or lung hypoplasia, platelet count of < 50.000/mm^3^, oliguria < 1 ml/kg/h, and absence of informed consent of the parents. All exclusion criteria were taken into account before the randomization and blood collection for the measurement of NT-proBNP.

The randomization was performed using a computer-generated random series of numbers. Participants were randomly assigned following simple randomization procedures (computerized random numbers) to one of the two treatment groups or the expectant management group. Determining whether a patient would be treated with ibuprofen/paracetamol or not (expectant approach) was done by reference to a statistical series based on random sample numbers; details of the series were known to the investigator. After obtainment of informed consent, the first echocardiographic evaluation was performed at a postnatal age between 24 and 72 h. When the transductal PDA measured more than 1.5 mm and a predominantly left to-right shunt was observed, the patient was randomised to either the medical treatment arm (ibuprofen/paracetamol) or the expectant PDA management arm. It is obviously not possible to perform a blinded study, since it is a difference in intention between the three patient groups, namely on one side the intention to medically treat and close a PDA with ibuprofen/paracetamol and on the other hand the intention to accept a PDA as a non-pathological phenomenon and not specifically aiming at active closure of the ductus arteriosus.

Twenty-seven newborns (52%) were treated with ibuprofen (*n* = 13) or paracetamol (*n* = 14) starting within the first 3 days of life. Ibuprofen was administrated rectally as a single dose of 20/10/10 mg/kg/day for 3 days; paracetamol was given intravenously, 15 mg/kg/dose every 6 hours for 3 days. Echocardiographic evaluation was performed at least 12 h after the last dose of the first course. If the DA was found to be closed, no further investigation or treatment was needed. DA was considered closed if it could not be visualized using colour Doppler imaging or if its transductal diameter was < 0.5 mm. If the DA was not closed, the second course of treatment was started at least 24 h after the third dose of the first course [14].

Expectant management was applied to 25 (48%) infants. These infants did not receive specific treatment, but clinical observation and follow-up were the same as for the babies who received ibuprofen or paracetamol.

The primary endpoint was the development of BPD/death. This composite variable was chosen because of the competing nature of these two events. BPD was defined as the need for oxygen or respiratory support at 36 weeks’ PMA and/or hospital discharge according to the consensus recommendations of the National Institutes of Child Health and Human Development in the modification of Walsh et al. [15, 16].

### Measurement of serum concentrations of NT-proBNP

The blood samples were collected twice: within the first 24–72 h of life, or before the administration of ibuprofen/paracetamol and after the end of the second course of the treatment, or at the eighth or ninth day of life if the expectant management was used. The median [IQR] age of the first and repeated blood sampling were two [1, 2] and eight [8, 9] days, respectively. After each blood collection, serum was immediately obtained, frozen, and stored at minus 25 °C. Serum NT-proBNP concentrations were measured using electrochemiluminescence immunoassay technique (Elecsys proBNP II test; Roche Diagnostics, Germany) according to the manufacturer’s recommendations. Available measurement limits were 5–35,000 pg/mL.

### Echocardiographic measurements

All patients underwent planned daily echocardiographic study within the first 10 days after birth, with a follow-up examination at 28 days of life and PMA of 36 weeks and/or discharge (whatever came first). Echocardiographic examination was performed using a Samsung Medison SonoAce X8 ultrasound machine (South Korea).

Ductal significance was determined based on a ductal diameter > 1.5 mm with unrestricted ductal left-to-right shunting (‘pulsatile pattern’): end-diastolic flow velocity < 50% of peak flow velocity; end-diastolic flow velocity > 0.3 m/s; and/or the ratio of the left atrial to aortic root diameter (LA/Ao) > 1.5; and/or the presence of retrograde diastolic blood flow in descending aorta. At least one of the following signs, such as deterioration of the respiratory status with the requirement for invasive ventilation, increase in oxygen dependency, inability to wean from respiratory support, systemic hypotension, or congestive heart failure had to be present to confirm ductal hemodynamic significance [17, 18].

### Clinical data and monitoring

Standard protocols of respiratory support, disease management, and nutrition, as well as routine vital signs monitoring, were applied to all newborns. Overall illness severity on the first day of hospitalization was standardly assessed with the SNAPPE-II scores [19].

### Statistical analysis

Receiver operating characteristic (ROC) curves were used to assess the ability of serum NT-proBNP concentrations to predict BPD/death, determining the area under it and calculating the corresponding sensitivity and specificity.

Standard methods of descriptive, comparative, correlation, and logistic regression analysis using the χ^2^, Mann-Whitney, and Wilcoxon criteria, as well as adjusted odds ratios (aOR), were used in the study. Nonparametric data are presented as a median [IQR]. Correlation between nonparametric variables was assessed with Spearman’s rank correlation coefficient (r_S_). All values were considered significant if *p* < 0.05.

## Results

### Comparative clinical characteristic of the groups

BPD was diagnosed in 19 infants (36%). In 14 of them (74%) the disease was mild, in 4 (21%) – moderate, and in 1 (5%) – severe. One child with mild BPD died of late sepsis. Overall, 7 infants died. The most common cause of death was severe intraventricular haemorrhage (IVH) (5 infants - 72%). There were no statistically significant differences in the frequency of pharmacological treatment of PDA between the infants who died of IVH (3 of them (60%) were treated with ibuprofen (*n* = 1) and paracetamol (*n* = 2) and 2 (40%) - did not receive specific treatment, *p* = 0.81). The median age of occurrence of IVH was 4 (3.5–5) days. One child died of NEC (14%). The median age at the time of death was 8.1 (7.2–15) days.

Infants who died or survived with BPD had significantly lower gestational age and birth weight, were more often delivered to mothers with clinical chorioamnionitis or fever (> 38° С) during labour (4 cases – 16% vs. 0 cases, respectively, *p* = 0.031), and more often had a complicated clinical course. The clinical characteristics of the groups are summarized in Table 1.

**Table 1:** Comparative clinical characteristic of the groups

Characteristics	BPD/death (*n* = 25)	Without BPD (*n* = 27)	*p*
***Demographic data and care after birth***			
Gestational age, wks	27 (25–28)^a^	30 (28–31)	***0.000001***
Birth weight, g	820 (740–1000)	1200 (950–1350)	***0.00003***
Male	16 (64)^b^	10 (37)	0.052
Small for gestational age	0	5 (19)	***0.023***
Antenatal steroids	15 (60)	21 (78)	0.165
Caesarean section	5 (20)	17 (63)	***0.002***
Apgar score at the 1st min	4 (4–5)	6 (5–6)	***0.001***
Apgar score at the 5th min	5 (5–6)	6 (6–7)	***0.001***
Mask ventilation after birth	4 (16)	3 (11)	0.605
Intubation and ventilation after birth	21 (84)	14 (52)	***0.014***
Surfactant therapy	24 (96)	19 (70)	***0.015***
SNAPPE-II score	34 (28–44)	18 (8–23)	***0.00001***
***Morbidity***			
Severe RDS			
Early-onset sepsis	12 (48)	6 (22)	0.051
Late-onset sepsis	4 (16)	1 (4)	0.132
Necrotizing enterocolitis	2 (8)	1 (4)	0.506
Intraventricular haemorrhage (IVH)	12 (48)	7 (26)	0.099
Severe IVH (3–4 grade)	7 (28)	1 (4)	***0.015***
hsPDA	17 (68)	5 (19)	***0.0003***
Duration of hsPDA, days	5 (3–12)	3 (3–4)	0.433
Pharmacological treatment	14 (56)	13 (48)	0.571
Successful pharmacological PDA closure	8 (57)	11 (85)	0.118
Two courses of treatment	6 (24)	3 (11)	0.471
The presence of PDA at 10 days of life	14 (56)	8 (30)	0.054
Age at the time of ductus closure, days	8 (5–15)	7 (5–11)	0.448
Ductus closed at the time of discharge	16 (64)	24 (89)	0.313
Periventricular leukomalacia	4 (16)	0	***0.031***
Retinopathy of prematurity	10 (40)	5 (19)	0.088
Length of hospital stay^c^, days	91 (79–112)	57 (45–71)	***0.000003***
***Respiratory support in the NICU***			
Mechanical ventilation (MV)	24 (96)	18 (67)	***0.007***
Non-invasive MV	20 (80)	7 (26)	***0.0001***
CPAP	21 (84)	27 (100)	***0.031***
Free-flow oxygen	18 (72)	12 (44)	***0.044***
Duration of MV^c^, days	2 (1–5)	0,9 (0,5–1)	***0.037***
Duration of non-invasive respiratory support^c^, days	19.6 (12.1–36.3)	7.4 (3.4–14.8)	***0.004***
Duration of oxygen therapy^c^, days	25.5 (21–38)	9 (2–13.1)	***0.00002***

^a^ median (interquartile range); ^b^ number of cases (%); ^c^ for infants who survived

The median size of PDA in the first 3 days of life was significantly smaller in infants who survived without BPD. At the same time, echocardiographic signs of hsPDA were significantly more often found in infants who died or developed BPD (Table 2). There were no statistically significant differences between the groups in the frequency of pharmacological treatment and requirements for the second course of treatment (Table 1). However, pharmacological treatment of hsPDA did not significantly reduce the incidence of BPD (9 infants (53%) versus 8 (47%) in infants who did not receive treatment, *p* > 0.05). At the same time, longer persistence of hsPDA did not increase the risk of death or future BPD development (r_s_ = 0.16, *p* = 0.47).

**Table 2:** Comparative characteristics of echocardiographic parameters of hsPDA in the first 3 days of life

Parameters	BPD/death (*n* = 25)	Without BPD (*n* = 27)	*р*
Ductal diameter, mm	2.5 (2.5–3.2)^a^	2.5 (1.8–3)	***0.031***
End-diastolic flow velocity < 50% of peak flow velocity	20 (80)^b^	12 (44)	***0.008***
End-diastolic flow velocity > 0.3 m/s	20 (80)	8 (30)	***0.0003***
Left atrium to aortic root diameter ratio > 1.5	12 (48)	1 (4)	***0.0002***
Retrograde diastolic blood flow in descending aorta	18 (72)	6 (22)	***0.0003***

Notes: ^a^ median (interquartile range); ^b^ number of cases (%)

Infants who developed BPD/died were more likely to require mechanical ventilation (MV), non-invasive ventilation, constant positive airway pressure (CPAP), and free-flow oxygen. The total duration of MV, non-invasive respiratory support, and oxygen therapy were nearly three times longer in infants who survived with BPD. The length of the hospital stay was almost twice as long in the infants who developed BPD (Table 1). The mean fraction of inspired oxygen (FiO_2_) at 8 days of life did not significantly differ between the groups (21 (21–35) % in infants who died or developed BPD, versus 21 (21–21) %) in infants who survived without BPD, *p* = 0.083).

### Serum NT-proBNP values

Both median NT-proBNP values were significantly higher in infants who died or developed BPD. By the eighth day of life, the NT-proBNP concentrations have significantly decreased in all infants but remained considerably lower in infants who survived without BPD (Table 3).

**Table 3:** Comparative serum NT-proBNP concentrations in the groups

Indicators	BPD/death (*n* = 25)	Without BPD (*n* = 27)	*р*
NT-proBNP level in the first 24–72 h of life, pg/ml	17,916 (8920-26,373)^a^	7391 (3257-16,868)	***0.008***
NT-proBNP level for 8–9 days of life, pg/ml	3988.5 (1839-8017)^b^	1717 (1284-2409)^b^	***0.002***

^a^ median (interquartile range); ^b^ the dynamics of the indicator is statistically significant (*p* < 0.01)

**Fig. 2:**
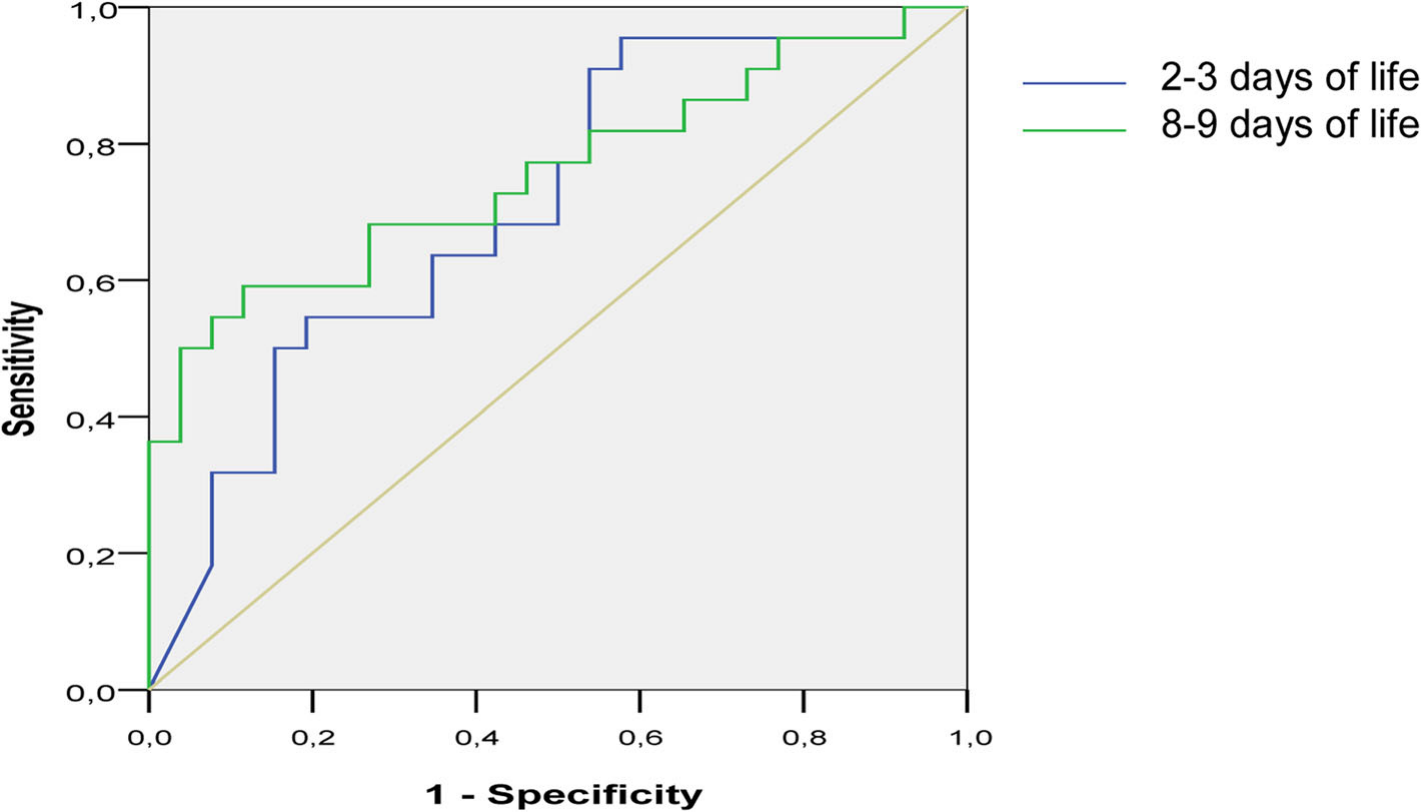
ROC-curves describing N-terminal pro-brain natriuretic peptide cut-off values for the prediction of BPD/death at the median age of 2 and 8 days in very preterm infants with PDA (the first measurement - 2-3 days of life; the second measurement - 8-9 days of life)

Based on the area under the ROC-curve (AUC) serum NT-proBNP levels on the 2–3 and 8–9 days of life could reliably predict death or BPD development in very preterm infants with PDA > 1.5 mm in the first 3 days of life. The prognostic value of this indicator was higher and more reliable at the age of 8–9 days of life. Thus, the AUC for 2–3 days of life was 0.71 (95% confidence interval (CI): 0.56–0.9, *p* = 0.014), and for 8–9 days of life – 0.76 (95% CI: 0.6–0.9, *p* = 0.002) (Fig. 2). Serum NT-proBNP concentrations ≥17,745 pg/ml at day two or three had 55% sensitivity and 81% specificity for the prediction of death or BPD. At the same time, serum NT-proBNP concentrations ≥3537 pg/ml at day 8 had 60% sensitivity and 89% specificity for the prediction of death or BPD.

Median NT-proBNP values in the first 3 days of life in infants who later developed BPD or died depended on the hemodynamic significance of PDA but it was the same for the infants who survived without BPD (Table 4). NT-proBNP values at 2–3 days of life were higher in infants with hsPDA who later died or developed BPD than in infants with hsPDA who survived without BPD, but the difference was only approaching statistical significance (*p* = 0.064).

**Table 4:** Serum NT-proBNP levels in the groups depending on haemodynamic significance and persistence of PDA

**Groups**	**hsPDA (*n* = 22)**	**Hemodynamically insignificant PDA (*n* = 30)**	*р*
***NT-proBNP values in the first 3 days of life***			
BPD/death	23,443 (17,916-35,000)	5968.5 (4757-8123.5)	***0.00007***
Without BPD	17,941 (15,920-24,220)	4027 (3230-12,620)	***0.008***
***NT-proBNP values at 8–9 days of age***			
Groups	Persistent PDA up to 10 days of life (*n* = 22)	Closed ductus by the tenth day of life (*n* = 30)	*p*
BPD/death	7019.5 (3988.5-29,134.5)	1805.5 (1305-3555)	***0.004***
Without BPD	3316 (2273.5-3772)	1558 (1053-1867)	***0.0006***

Median (interquartile range)

Median NT-proBNP values at 8–9 days of age in infants who died or developed BPD depended on the presence of persistent PDA up to 10 days of life (Table 4). In infants who survived without BPD, median NT-proBNP concentrations at 8–9 days of age were also associated with the PDA persistency during the first 10 days of life (Table 4). Median NT-proBNP values at 8–9 days of age were significantly higher in infants with persistent PDA who died or developed BPD than in babies with persistent PDA who survived without BPD (*p* = 0.014).

Serum NT-proBNP values at 8–9 days of life were also associated with FiO_2_ at 8 days of life (r_S_ = 0.46, *p* = 0.01). Serum NT-proBNP levels at median ages of 2 and 8 days were significantly higher in neonates with a lower gestational age (r_S_ = − 0.37; *p* = 0.008 and r_S_ = − 0.41; *p* = 0.004, respectively).

According to the results of multivariate logistic regression analysis, which included the gestational age, for every one-unit increase in the natural logarithm of the concentration of NT-proBNP in the first 3 days of life, the odds of death or BPD increased almost 3-fold (aOR = 2.613; 95% CI: 0.901–7.578; *p* = 0.077), and at 8–9 days of life this increase was almost 5-fold (aOR = 4.64; 95% CI: 1.197–17.993; *p* = 0.026).

## Discussion

hsPDA is a common complication in very preterm infants born at < 32 weeks of gestation. It is known that a large left-to-right PDA shunt significantly increases the risk of developing BPD or death [2, 3]. The results of this study showed that infants who died or developed BPD significantly more often had hsPDA than babies who survived without BPD. Our data confirmed the results of a recent meta-analysis [13], according to which in five out of nine studies the hsPDA or PDA was significantly more likely diagnosed in infants who developed BPD. According to our results, death or BPD occurred significantly more often in infants with lower gestational age, birth weight, and the condition of such infants after birth was more compromised, as evidenced by significantly lower Apgar scores, the need for intubation and MV in delivery room as well as by the significantly higher SNAPPE-II scores on the first day of NICU hospitalization.

The median size of PDA in the first 3 days of life was significantly smaller in infants who survived without BPD. Echocardiographic markers of hsPDA at this age were significantly more often seen in infants who died or developed BPD. At the same time, according to our results, pharmacological treatment of hsPDA did not significantly reduce the incidence of BPD. Therefore, despite the association between PDA and BPD/death in epidemiological studies, screening and early treatment of infants with PDA in recent years have not reduced the risk of BPD, suggesting that the relationship between PDA and BPD may not be causal [20]. In particular, the association between hsPDA and severe neonatal morbidity differs in different studies, remaining one of the most controversial aspects of the problem [21, 22].

It is now known that there is a dose-dependent correlation between the magnitude of the PDA shunt and the risk of BPD. El-Khuffash et al. [23] proposed a PDA score based on echocardiography measurements at 48 h after birth (*n* = 141). Their results showed that gestational age, together with echocardiographic markers of PDA and left ventricular diastolic function, may predict the later occurrence of BPD or death in preterm infants < 29 weeks of gestation. A PDA score cut-off of 5 or more had sensitivity and specificity of 92 and 87%, and the positive and negative predictive values of 92 and 82%, respectively [23]. Thus, the relationship between PDA and BPD strengthens with decreasing gestational age and increasing ductal flow. At the same time, the duration of PDA exposure can also play an important role in the development of BPD. Schena et al. [2] assessed PDA significance using staging system proposed by McNamara and Sehgal [24] in infants < 29 weeks of gestation (*n* = 242). According to their results, the risk of developing BPD increased by 70% for each additional week of exposure to the hsPDA. A recent study by Mirza et al. [25] also showed that the longer duration of hsPDA in extremely preterm infants was associated with a higher risk of death or BPD development (aOR = 1.37; 95% CI 1.03–1.82). However, our results did not prove the association between the duration of hsPDA and the risk of death or BPD development.

One of the clinical predictors of BPD development is a pattern of oxygen dependence in the first 2 weeks of life [26]. The level of oxygen dependency in our patients on the eighth day of life was not associated with later BPD development, possibly due to the predominance of mild BPD in our patients. At the same time, the results of our study showed that serum NT-proBNP values at 8–9 days of life were significantly associated with FiO_2_ at this age.

The results of lung ultrasound have high diagnostic value in predicting the risk of developing BPD, but only after 1 week of life [27]. Despite the diagnostic value of echocardiography and lung ultrasound, the interpretation of the results of both these examinations is subjective, requires involvement of skilled specialists, and the examination itself requires additional costs. Determination of biomarkers allows to eliminate the subjectivity, does not require additional equipment and specialists, and thus could be cheaper and more practical. The relationship between hsPDA and the risk of developing BPD is known, but the mechanism of this association remains poorly understood and requires further investigation.

The prognostic value of potential BPD biomarkers has not been sufficiently studied, as the feasible and reliable biochemical indicator that could accurately predict the development of BPD in the first days of life in very preterm infants has not yet been identified [10]. It is known that systemic NT-proBNP concentrations have a high sensitivity and specificity for predicting the hsPDA in the first days of life [11]. Serum NT-proBNP concentrations ≥17,745 pg/ml in our patients at a median age of 2 days had 55% sensitivity and 81% specificity for predicting BPD or death. Our data are consistent with the results of a study by Sellmeer & Singer [17], which showed that the serum NT-proBNP concentrations on the third day of life could reliably predict the development of BPD/death in infants < 32 weeks of gestation (AUC = 0.76; 95% CI: 0.68–0.84). At the same time, their results, like ours, showed that in infants with hsPDA who later developed BPD or died, the serum NT-proBNP levels at 3 days of life were significantly higher than in infants with hemodynamically insignificant or closed ductus [17].

According to the results of our study, the median NT-proBNP value at 8–9 days of life was not only associated with PDA persistency up to 10 days of life but also reliably determined the risk of BPD or death. Serum NT-proBNP concentrations ≥3537 pg/ml at 8–9 days of life had 60% sensitivity and 89% specificity for the prediction of BPD or death regardless of the PDA persistency till the tenth day of life. A similar conclusion was reached by Rodríguez-Blanco et al. [12], who compared the NT-proBNP level at 48–96 h of life and 5–10 days after birth in preterm infants ≤32 weeks of gestation (*n* = 110). The authors demonstrated that the relationship between NT-proBNP concentration at 48–96 h of life and development of BPD or death is weak and probably confounded by the presence of hsPDA. However, the NT-proBNP level at 5–10 days of life could reliably predict BPD or death independently from the presence of hsPDA (OR = 3.36, 95% CI: 1.52–7.4, *p* = 0.006). The NT-proBNP concentration > 3348 pg/ml at 5–10 days of life was the best cut-off value for predicting BPD/death with 82% sensitivity and 83% specificity (AUC = 0.87; 95% CI: 0.79–0.95) [12]. According to the results of Harris et al. [28], even lower NT-proBNP concentration > 1598.3 pg/ml on the tenth day of life had 84% sensitivity and 75% specificity in predicting severe BPD (AUC = 0.83) in infants < 30 weeks of gestation (*n* = 51). At the same time, in a study by Zhou et al. [29] the serum NT-proBNP concentration > 2002.5 pg/ml on the first days of life was predictive for the development of moderate/severe BPD or death with 87.5% sensitivity and 74.7% specificity (AUC = 0.85, 95% CI: 0.79–0.91). Therefore, currently available evidence demonstrates that serum NT-proBNP concentration in very preterm infants in the first 10 days of life could reliably predict the development of BPD or death with high sensitivity and specificity regardless of the presence of PDA.

The advantages of our study are the prospective design and randomized treatment of very preterm infants with PDA diameter > 1.5 mm in the first 3 days of life, that allowed to eliminate the subjective factor in making management decisions, and objectively assess the predictive value of systemic NT-proBNP concentrations for the development of BPD or death in infants with PDA. Our results also indicate that early NT-proBNP screening could reduce the workload and resource utilization associated with PDA assessment in very preterm infants, especially in settings with limited access to around-the-clock echocardiography expertise, and significantly limit the presence of subjectivity component within that assessment. The estimated time required to perform an echocardiographic assessment of the patient significantly exceeds the duration of blood sampling. Additionally, the cost of echocardiographic examination is 10 times higher than the cost of NT-proBNP level determination. Our study did not involve any specific analysis of the economic side of this problem, but obtained data may suggest that NT-proBNP screening could reduce the overall financial burden associated with PDA screening.

Our study also has some limitations, primarily due to its non-blinding design, specific approach to treatment (early administration based only on the PDA size), relatively small number of patients, and proportion of the most immature infants. Prospects for further research are to study the possibility of using serum NT-proBNP concentrations in the first days of life to form a risk group and targeted testing of potential preventive interventions.

## Conclusions

In very preterm infants with PDA > 1.5 mm at the age of 24–48 h, serum NT-proBNP concentration could reliably predict the development of BPD or death, regardless of the persistence of PDA, with the highest diagnostic value at 8–9 days. The measurements ≥17,745 pg/ml at the age of 24–48 h had 55% sensitivity and 81% specificity for predicting BPD/death, and concentrations ≥3537 pg/ml on the 8–9 days of life – 60 and 89%, respectively.

Regardless of later death or development of BPD, the serum NT-proBNP values at 2–3 days of life were higher in very preterm infants with hsPDA, and at 8–9 days of life – in babies with persistent PDA.

hsPDA was significantly more often diagnosed in infants who later developed BPD or died, however, pharmacological treatment of hsPDA did not reliably reduce the incidence of BPD or death.

## Data Availability

The datasets used and/or analysed during the current study available from the corresponding author on reasonable request.

## Abbreviations

BPD: Bronchopulmonary dysplasia
CPAP: Constant positive airway pressure
FiO2: Fraction of inspired oxygen
hsPDA: Hemodynamically significant patent ductus arteriosus
IVH: Intraventricular haemorrhage
LA/Ao: Left atrium to aortic root diameter ratio
MV: Mechanical ventilation; NEC: Necrotizing enterocolitis
NICU: Neonatal intensive care unit
NT-proBNP: N-terminal pro-brain natriuretic peptide
PDA: Patent ductus arteriosus
PMA: Postmenstrual age
